# Jump ahead with a twist: DNA acrobatics drive transposition forward

**DOI:** 10.1016/j.sbi.2019.08.006

**Published:** 2019-12

**Authors:** Vladimir Arinkin, Georgy Smyshlyaev, Orsolya Barabas

**Affiliations:** 1 Structural and Computational Biology Unit, European Molecular Biology Laboratory, Heidelberg, Germany; 2 European Molecular Biology Laboratory, European Bioinformatics Institute (EMBL-EBI), Wellcome Genome Campus, Hinxton, UK

## Abstract

Transposases move discrete pieces of DNA between genomic locations and had a profound impact on evolution. They drove the emergence of important biological functions and are the most frequent proteins encoded in modern genomes. Yet, the molecular principles of their actions have remained largely unclear. Here we review recent structural studies of transposase-DNA complexes and related cellular machineries, which provided unmatched mechanistic insights. We highlight how transposases introduce major DNA twists and kinks at various stages of their reaction and discuss the functional impact of these astounding DNA acrobatics on several aspects of transposition. By comparison with distantly related DNA recombination systems, we propose that forcing DNA into unnatural shapes may be a general strategy to drive rearrangements forward.

## Highlights

• Transposition reactions are ubiquitous and involve complex DNA rearrangements.
• Transposase-DNA complex structures reveal key mechanistic principles.
• DNA bending, unwinding and melting promote all steps of transposase function.
• DNA distortions serve universal roles in transposition and related cellular processes.

**Current Opinion in Structural Biology** 2019, **59**:168–177

This review comes from a themed issue on **Protein nucleic acid interactions**

Edited by **Frédéric H-T Allain** and **Martin Jinek**

For a complete overview see the Issue and the Editorial

Available online 5th October 2019

**https://doi.org/10.1016/j.sbi.2019.08.006**

0959-440X/© 2019 The Authors. Published by Elsevier Ltd. This is an open access article under the CC BY license (http://creativecommons.org/licenses/by/4.0/).

## Introduction

Transposable elements (TEs) are discrete segments of DNA that can move from one location to another in genomes. They are abundant across the tree of life [1,2], and their movement has shaped evolution, driving genetic variation, horizontal gene transfer, genome remodeling, and the emergence of distinct regulatory networks [3,4]. Several TEs have been ‘domesticated’ to provide important cellular functions in their host organisms, with prime examples including the V(D)J recombination system responsible for antibody diversification in vertebrates [5] and programmed DNA rearrangements involved in somatic genome assembly in ciliates [6]. Furthermore, TEs have been exploited to provide tools for functional genomics, sequencing, transgenesis, stem cell engineering, and gene therapy applications [7, 8, 9, 10, 11]. Depending on their mechanisms, TEs are divided in two major classes: DNA transposons that move using only DNA intermediates and retrotransposons that employ RNA intermediates. In this review we focus on the structural principles of DNA transposons; for comprehensive reviews of retrotransposons and specific DNA transposon types we refer the reader to chapters of Mobile DNA III [12].

**Figure 1:**
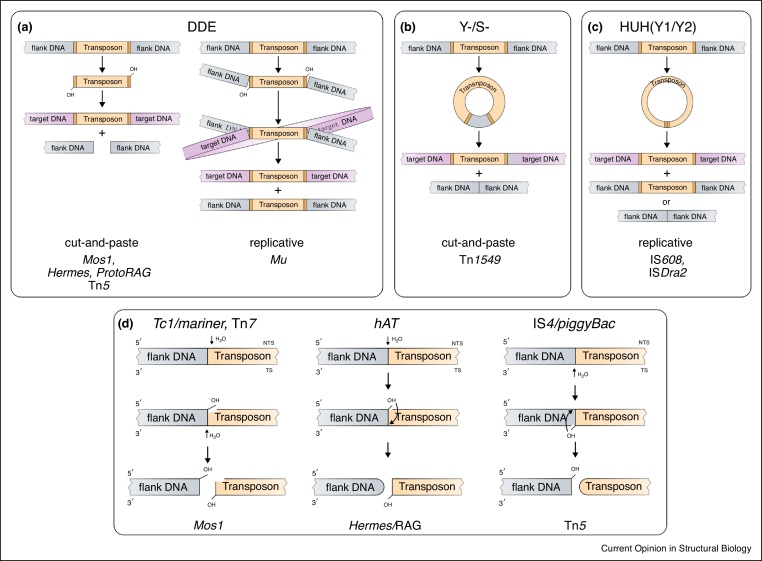
Transposition pathways catalyzed by DNA transposases. **(a–c)** Schematics of the main steps of transposon excision and integration in distinct transposase families. Examples for which high-resolution transposase-DNA complex structures are available are listed at the bottom. The color scheme (beige: transposon DNA; orange: transposon ends; grey: flanking donor DNA; violet: target DNA) is retained throughout. (a) Main pathways used by DDE transposases. In the cut-and-paste process, the transposon is excised from its original location through DNA double strand breaks. Integration occurs by attack of the liberated 3′-OH groups on a target DNA. In replicative transposition, the element is only nicked on both ends and integration creates a so-called Shapiro intermediate. This is then resolved by replication, generating a new transposon copy at the target site. Some transposases combine features of these main routes, for example, utilizing replication to proceed via excised circular intermediates. (b) Transposition by Y-transposases and S-transposases. Excision creates a double-stranded circular intermediate with the transposon ends abutted. Y-transposases enclose a short stretch (5–7 base pairs) of flanking DNA between the ends. The donor DNA is simultaneously resealed. Recombination of the transposon circle with target DNA, usually in a new bacterial cell, leads to integration. (c) Pathway of HUH-like (Y1-/Y2-) transposases. A single-stranded transposon DNA circle is excised and integrated. Replication re-generates the second DNA strand. **(d)** Schemes of double strand DNA cleavage in DDE enzymes. The DNA strand that contains the 3′-OH on the transposon end used for subsequent integration is denoted as transferred strand (TS); the complementary strand is labeled non-transferred strand (NTS). The TS is cleaved always precisely at the transposon end, while the site of NTS cleavage varies. Adapted from [13].

DNA transposons vary in size from a few hundred to a hundred thousand base pairs. They contain specific DNA sequences at their ends, which generally enclose one or more protein-coding genes. Autonomous TEs encode at least one enzyme, the transposase, which recognizes the transposon ends and catalyzes DNA cleavage and joining reactions required for their movement (transposition). Some TEs additionally encode accessory proteins that support distinct transposition steps or carry genetic cargos such as antibiotic resistance genes. Although conceptually similar, DNA transposons follow various molecular pathways (Figure 1) [13,14]. Many elements move by a ‘cut-and-paste’ process, where DNA is cleaved at the two transposon ends and then inserted into a new genomic location. Others undergo ‘replicative’ transposition, where the transposase nicks a single DNA strand at each transposon end and replication creates a copy of the element at the new site, while leaving the original copy preserved at its old location (Figure 1a–c).

To execute diverse transposition pathways, a variety of structurally and mechanistically distinct transposase enzymes have emerged. All these possess DNA binding and nuclease activities, but vary greatly in their fold, domain composition and chemistry [13]. A large group of transposases, known as DDE transposases, cut DNA using an RNase H-like catalytic domain. These contain a conserved triad of acidic residues (usually DDE), which coordinate two divalent metal ions to activate a nucleophile water or a hydroxyl group to attack a phosphorous atom in the DNA (Figure 1a). Y-transposases and S-transposases that are related to site-specific recombinases employ a tyrosine or serine residue in their active sites and become covalently attached to the DNA upon cleavage (Figure 1b). HUH nuclease-like transposases (Y1-transposases and Y2-transposases) also pass through a covalent protein-DNA intermediate with an active site tyrosine, but involve single-strand DNA intermediates (Figure 1c).

Although seemingly simple, transposition reactions require a series of DNA binding, cleavage and rearrangement events. At least two DNA sites must be recognized at the transposon ends, followed by stepwise cleavage of four DNA strands to excise the element. Then, a target DNA substrate of variable sequence must be captured and prepared for transposon insertion. Remarkably, all these DNA rearrangements can be carried out by relatively small (15–170 kDa) transposase enzymes, without the need for high energy co-factors. However, the mechanisms are still incompletely understood. Despite great efforts, structural insights into active transposition assemblies are still sparse and mechanistic models often rely on predictions from distantly related systems. Here we review recent studies of transposase-DNA complexes, which provided key insights into the structural basis of transposition. Interestingly, these works highlight a re-occurring role of major DNA distortions, twists and kinks in promoting several aspects of transposition. We discuss the molecular principles of this emerging phenomenon, its impact on diverse transposition steps, and suggest that exercising DNA acrobatics may be a common feature of mobile DNA and related cellular systems.

## Start with a twist — DNA unwinding promotes cleavage initiation

DDE transposases initiate transposition by hydrolyzing one DNA strand on each transposon end, creating a 5′-phosphate and a 3′-hydroxyl group at the cleavage site (Figure 1d). In cut-and-paste transposition, the complementary DNA strand is then also cleaved, either using a second water molecule (e.g. in Tn*7* and *Tc1/mariner* elements) or by attack of the 3′-OH group of the first cleaved strand. Depending on the site of first cleavage, the latter pathway creates a hairpin intermediate on the excised transposon (e.g. IS*4*, *piggyBac*) or on the flanking DNA (*hAT* elements and V(D)J recombination) [13] (Figure 1d). Hairpins on the transposon must then be reopened, and integration follows by attack of the free 3′-OH groups of the transposon ends at a new genomic site.

Early structures of DDE transposase-DNA complexes revealed intertwined assemblies, where two transposon ends are synapsed by a transposase dimer. In this synaptic complex or transpososome, each transposase molecule interacts with both transposon ends [15, 16, 17], ensuring that reaction can only occur once the transposase located both transposon ends. However, these structures captured excised transposon intermediates, and the molecular arrangements involved in initial DNA binding and cleavage have remained unknown.

First insights into the initiation of transposition have recently emerged from studies of the *Hermes* transposase from the *hAT* superfamily [18^••^], the RAG enzyme complex (RAG) from the V(D)J system [19^•^,20^••^,21^•^] and its closest transposase relative ProtoRAG [22^••^]. RAG is a domesticated transposase that initiates V(D)J recombination in the adaptive immune system, mediating synapsis and cleavage at recombination signal sequences (RSS, the transposon end equivalent) to promote combinatorial assembly of adjacent V, D and J antibody-encoding gene segments (the coding flank, equivalent to the transposon flanking DNA) [23]. Both Hermes and RAG cut the ‘non-transferred’ DNA strand first and use the liberated 3′-OH to attack the opposite (transferred) strand, generating a hairpin on the flank (Figure 1d).

**Figure 2:**
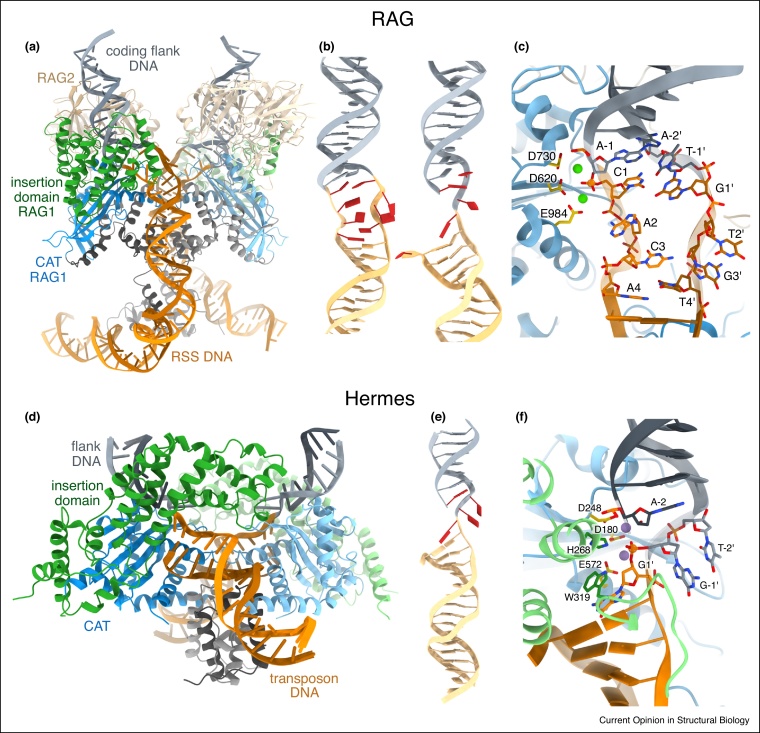
DNA distortion promotes cleavage in RAG and the Hermes transposase. **(a–f)** Transposase-DNA complex structures are shown with the RNase H-like catalytic domain (CAT) in blue, the insertion domain in green and other domains in grey. DNA is colored analogous to Figure 1, with the RSS/transposon ends in orange and the flank in grey. (a) Cryo-EM structure of the RAG-DNA complex before first strand cleavage (PDB: 6DBL). Two RAG1-RAG2 protein pairs synapse two intact DNA substrates (colored in different shades). RAG1 is colored as above, RAG2 is beige. (b) The shape of intact unwound (left) and nicked (right) DNA in the RAG structures (PDB: 6DBL and 6DBI, respectively). DNA nucleotides involved in the distortions are highlighted in red. (c) Close-up of the RAG active site with intact unwound DNA. Two calcium ions (green balls) and the catalytic DDE triad (sticks) assemble around the scissile phosphate (orange sphere). Distorted DNA segments (as in B) are shown as sticks. (d) Crystal structure of Hermes with a nicked DNA substrate arranged for hairpin formation (PDB: 6DWW). Two DNA molecules are bound in a transposase dimer (colored in different shades). DNA is sharply kinked at the boundary of transposon and flank. (e) Cartoon of the Hermes DNA, with flipped-out or unpaired nucleotides shown in red. (f) Close-up of one Hermes active site. The catalytic residues (DDE) are shown as sticks in yellow. Two manganese ions (purple balls) interact with the scissile phosphate (orange sphere). Deformed DNA parts are shown as sticks and transposase residues from the insertion domain that help shape the DNA for hairpin formation are in green.

Cryo-EM structures of RAG complexed with intact DNA substrate showed that initial DNA binding involves protein-mediated bending at several pivot points (with a total of 60–150° net curvature; Figure 2a) [19^•^,20^••^,21^•^]. Steric constraints with the coding flank DNA invoke opening of the protein (compared to its apo state), especially involving the ‘insertion domain’ that interrupts the canonical RNase H-like fold. This may create a strain that induces a backward movement of the protein and triggers a corkscrew-like 180° rotation of the DNA coding flank, with marked unwinding, melting and base flipping at the RSS-flank junction (Figure 2a,b) [20^••^]. This movement results in the formation of entirely new protein contacts with the coding flank, while the RSS DNA and the RNase H active site remain stationary. Importantly, the DNA distortion places the scissile phosphate in the catalytic center (Figure 2c), thus enabling precise cleavage at the RSS-flank boundary [20^••^]. Structures with nicked DNA intermediates revealed an overall similar DNA shape (Figure 2b), confirming that chemistry can proceed without further architectural changes [19^•^,21^•^]. Analogous structures of the ProtoRAG [22^••^] and Hermes [18^••^] transposases also reflected similar DNA shapes, consistent with an overall conservation of their excision pathways. Additionally, the structures showed that conserved sequences at the transposon ends (or RSS) are not engaged in protein binding, but rather play a role in facilitating DNA deformations. Specific pyrimidine-purine tracts at the transposon-flank boundary, which have a higher propensity to unwind and melt [18^••^,20^••^,^24^], allow the DNA to swivel more freely during excision. Flexible CA and TG dinucleotide sequences are also prevalent at the termini of other transposons [25], suggesting that DNA twisting may be a general feature of transposition initiation [20^••^]. This idea is also supported by biochemical experiments showing strong DNA distortion at flank-transposon junctions [26,27] and by post-cleavage transposase-DNA complex structures [15, 16, 17] pointing to a need for DNA reconfiguration during excision.

## Release with a kink — sharp ssDNA bending completes excision

Compared to the intact DNA complex, structures of RAG [19^•^,21^•^] and Hermes [18^••^] with nicked DNA revealed a more compact architecture with important changes in the active sites (Figure 2). Whereas binding of the flank DNA remained unchanged, with its liberated 3′-OH kept in the active site, the RSS/transposon DNA has shifted 5–6 Å relative to the flank axis (Figure 2b,e). The uncleaved strand showed a sharp kink, with several bases at the junction unpaired, unstacked and/or flipped out of the helical axis. This distortion placed the scissile phosphate of the second DNA strand near the 3′-OH in the active site in a proper configuration for hairpin formation (Figure 2f). A series of elegantly designed Hermes structures with different metal ions and DNA variants further elucidated the molecular basis of the DNA deformation [18^••^]. The structures revealed that the catalytic metal ions and amino acid residues from the transposase insertion domain cooperatively promote kinking of the DNA sugar-phosphate backbone. Several nucleotides become unpaired and the first two bases in the flank flip out from the DNA duplex, creating a conformation that resembles the hairpin product shape [18^••^] (Figure 2f).

Collectively, the structures showed that transposase active sites exhibit limited conformational changes during transposon excision. Instead, extensive DNA distortions bring the different scissile phosphates into the catalytic center to execute sequential cleavage and joining of distinct DNA strands. Specific DNA sequences at the transposon-flank boundary also play a role in facilitating the deformations: flexible pyrimidine-purine base steps allow DNA to unwind and melt [24], and specific interactions of flipped-out bases stabilize the distorted DNA states [18^••^,19^•^]. Interestingly, studies of *Tc1*/*mariner* transposases demonstrated a similar role of unpaired bases in positioning the transferred strand for accurate cleavage [28,29], thus extending the concept of ssDNA deformation to distant DDE enzymes.

## Stretch with a bend — target DNA bending drives integration

After excision, DDE transpososomes contain two transposon ends with free 3′-OH groups prepared for integration in a suitable target DNA (Figure 1a,d). Our understanding of how target DNA is bound and shaped in transpososomes is still limited. Previous target capture complex (TCC) structure of the distantly related retroviral integrase from the prototype foamy virus (PFV) revealed that target DNA binds in a conserved cleft between two integrase catalytic domains in a bent conformation [30]. PFV, Rous sarcoma virus (RSV) and human immunodeficiency virus (HIV) integrase structures with integration product DNA — containing one strand of each viral DNA end joined to the target (strand transfer complex, STC) — revealed a similar target bend [30,31,60].

**Figure 3:**
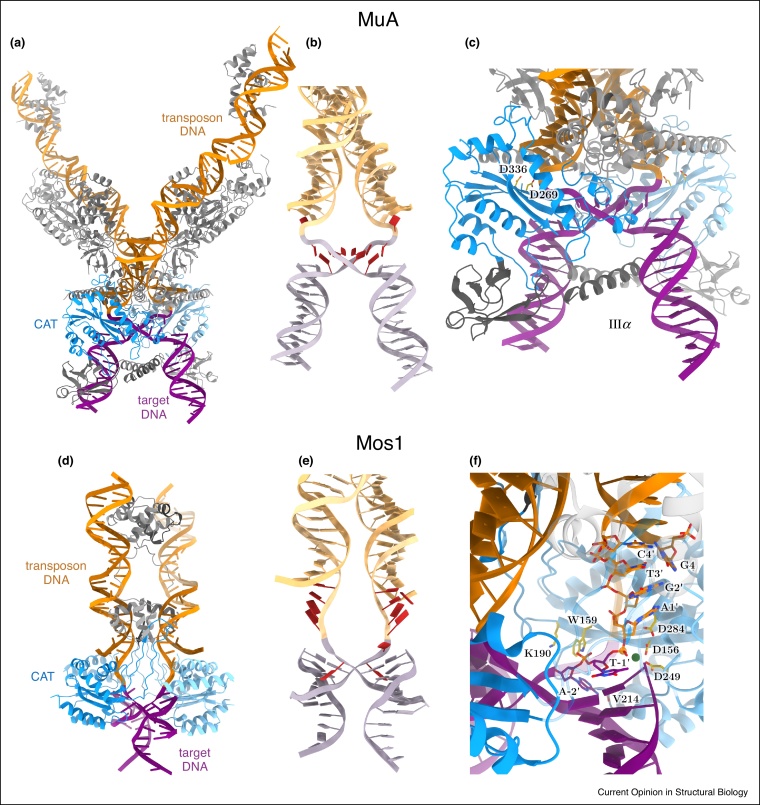
Target DNA bending is required for integration in the Mu and Mos1 transpososomes. **(a)** Crystal structure of the Mu strand transfer complex (PDB: 4FCY). Four MuA molecules bind the X-shaped DNA product of integration. Catalytic domains of the two active MuA subunits are colored in shades of blue, other domains and the accessory protein subunits are grey. Transposon end DNA is orange and target DNA is violet. **(b)** The DNA shape with nucleotides in distorted parts shown in red. **(c)** Close-up view of one active site. Catalytic residues are shown as yellow sticks. The coiled-coil domain (IIIα) on the concave side of the bent target is labeled. **(d)** Crystal structure of Mos1 strand transfer complex (PDB: 5HOO). Two transposase molecules bind the integration product DNA (colored as in a). **(e)** Cartoon of the DNA alone. **(f)** Close-up of the transposase active site. Unpaired and flipped nucleotides, catalytic residues and amino acids that stabilize base flipping are shown as sticks. The magnesium ion is green and the scissile phosphate is marked with an orange sphere.

Consistently, recent structures of transposase STCs reflected sharply bent target DNA. The transposase of bacteriophage Mu, which has served as a model for transposition for decades, bends its target by ∼140° [32^•^]. This impressive bend is promoted by extended interactions along the DNA backbone and by a C-terminal coiled-coil domain that dampens electrostatic repulsion between the target DNA arms (Figure 3a–c). A similarly sharp kink (147°) was observed in the recently determined STC structure of the *Tc1/mariner* family Mos1 transposase (Figure 3d,e) [33^••^], indicating that target DNA bending is a reoccurring theme in transposition. Remarkably, comparison of the Mos1 STC with the post-excision complex [16] revealed equivalent protein and transposon DNA arrangement, implying that target binding and integration occur without major changes in the rest of the complex [33^••^]. Thus, target DNA bending appears to have two essential functions. It brings the appropriate scissile phosphates into the preassembled transposase active sites, allowing the pre-arranged transposon 3′-OH groups to attack and join the target (Figure 3c,f). Additionally, the bending might strain the DNA such that it snaps away from the active sites after integration, rendering integration irreversible. Such product escape has been noted in all STC structures to date [30,31,32^•^,33^••^,^60^], implying that it is common in retroviral and transposon systems. Notably, insertion of the two transposon/viral ends occurs with different spacing in different systems (ranging from 2 to 9 bps), requiring markedly different degrees of target DNA bending. Inserting on the two sides of a TA/AT dinucleotide pair, *Tc1*/*mariner* transposases require the strongest target deformation, although the forces stabilizing the sharp bend are not entirely clear (Figure 3d–f).

As discussed for transposon ends, specific sequence features of integration sites likely promote target bending. Intrinsically flexible pyrimidine-purine base steps are enriched in insertion sites of most transposase and integrase families [33^••^,^34^] and biochemical studies showed that flexible, bent or mismatched sites are better targets for integration [35,36,37^••^,^38^]. Consistently, structural models of Tn*5*/Tn*10* [15,35], *Sleeping Beauty* [39] and *Hermes* [17] transpososomes revealed that only bent target DNA can fulfil the spatial requirements of staggered integration, further supporting the need for significant target bending in diverse systems.

## Attract with a flip — base flipping for integration site selection

Most transposases bind target DNA with low sequence specificity, allowing them to insert randomly throughout the genome. This increases their chance to re-integrate, and thus survive and propagate in genomes. For some elements (e.g. Tn*7* and Mu), partner proteins can direct integration to certain genomic regions, but the integration site specificity of the transposase itself is limited in all elements to a few base pairs. Preferred target sequences are generally palindromic and rich in T/A pairs, suggesting that their propensity for distortion is an important factor. However, several DDE transposases require invariable T/A nucleotides at distinct target positions, implying the need for specific recognition of some bases during integration.

In agreement, crystal structure of the Mos1 STC revealed direct interaction with the adenine bases in its strictly conserved TA/AT dinucleotide integration site [33^••^]. The adenine flips out into extra-helical space and forms base-specific contacts with the transposase main chain (V214). The deformed DNA backbone is stabilized via salt bridges and hydrogen bonds with specific protein residues (Figure 3f). Accordingly, time-resolved fluorescence and transposition assays confirmed an active role of DNA base flipping and backbone restructuring in TA recognition, target binding and integration [33^••^]. Similar distortions may be involved in recognition of conserved target positions in other DDE transposases, such as the TTAA tetranucleotide sites of *piggyBac* transposases or the preferred nTnnnnAn sequence of *Hermes*.

## Open to spread — DNA melting enables promiscuous integration

**Figure 4:**
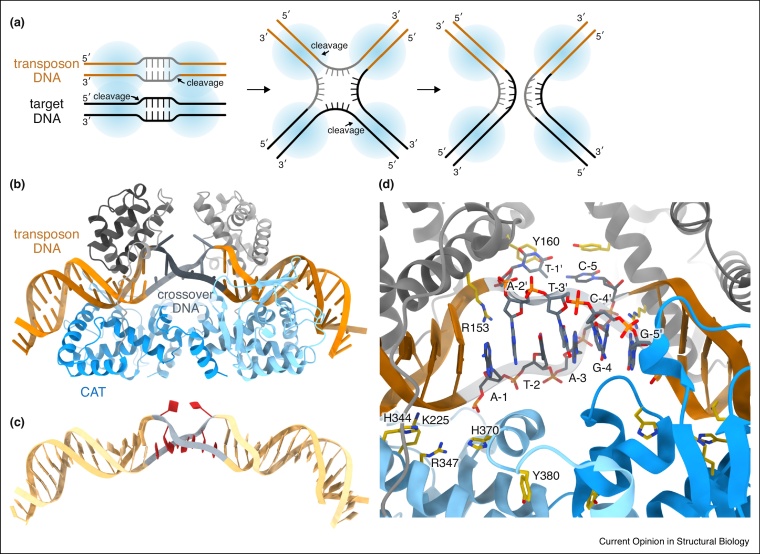
DNA melting enables conjugative transposons to insert at diverse genomic sites. **(a)** Proposed pathway for transposon circle (orange, grey) integration into target DNA (black) by Y-transposases (blue circles). Four DNA strands are cut and exchanged in pairs, proceeding through a four-way Holliday junction (HJ) intermediate. The DNA region that connects the transposon ends in the circle (grey) originates from the donor site and its sequence is different from the target. Thus, strand exchange creates a HJ with no base pairing in the center. **(b)** Structure of the Tn*1549* transposase (Int) with a circular transposon DNA intermediate mimic (PDB: 6EMZ). Two protein molecules (different shades) bind at the transposon ends (orange) with the melted crossover region in the middle (grey). Core DNA binding domain is grey and the catalytic domain is blue. **(c)** Cartoon of the DNA alone, with nucleotides in the distorted part in red. **(d)** Close-up of the Int active site. The unwound and melted DNA segments, and the residues involved in catalysis (including the nucleophile tyrosine) or base flipping are show as sticks.

Previous sections focused on DNA transposases with RNase H-like nuclease domains; yet, some major transposon groups employ completely unrelated transposase proteins (Figure 1b,c). For instance, conjugative transposons (also referred to as integrative conjugative elements, ICEs), which propagate antibiotic resistance genes in bacteria, utilize Y-transposases. These are related to site-specific tyrosine recombinases (TRs) [40,41] and catalyze DNA cleavage and joining at transposon ends without introducing double strand breaks [42]. First, they recombine DNA sequences at the two transposon ends to generate a covalently joined circular transposon intermediate. Integration then follows by recombination of the excised circle with a suitable target DNA, connecting all strands to the new genomic site (Figure 1b). By analogy with canonical TRs, such as the lambda phage integrase and the Cre recombinase, Y-transposases are thought to assemble two DNA molecules in a protein tetramer, where they cut and exchange DNA strands in pairs using a conserved tyrosine nucleophile, proceeding through a characteristic four-way Holliday junction (HJ) intermediate (Figure 4a) [41].

Previous structures of TRs demonstrated that DNA distortions play important roles in their functions [43, 44, 45, 46]. They bend DNA substrates (by ∼60–80°) at the cleavage sites to promote nicking and unpairing of the exchanged strands [47]. Bending also shapes the DNA arms such that their arrangement resembles the HJ, allowing easy transition between the reaction intermediates (Figure 4a) [46,48]. This necessity of DNA bending is likely preserved in Y-transposases, as they work through similar chemistry and DNA intermediates [49^••^,^50^,^51^]. However, their DNA substrates are fundamentally different. While most TRs recombine homologous DNA sites, Y-transposases have variable DNA substrates, especially for integration, where they insert at random sites in diverse bacterial genomes.

Remarkably, our recent structures of the vancomycin resistance-carrying Tn*1549* transposon revealed an unanticipated DNA deformation [49^••^]. The structures capture the transposase bound to circular transposon DNA mimics before the integration step (Figure 4b). Two transposase molecules bind symmetrically at the transposon ends, clamping DNA between their core DNA binding and catalytic domains. The arrangement of the proteins forces the DNA to distort, provoking a marked unwinding at the center (Figure 4c). A strategically placed arginine residue invades the DNA double helix, flipping out the terminal bases of the crossover region (Figure 4d). This opening of the duplex facilitates melting of the complementary DNA strands at the region where they will be swapped with the integration target, helping to overcome the energy barrier for strand exchange. Due to this integral DNA melting, the transposase can easily recombine DNA substrates regardless of their homology and thus execute integration into almost any target sequence [49^••^].

Sequence analysis also revealed that the protein residues required for DNA melting are conserved in the Tn*916*-like conjugative transposon family, indicating that the mechanism of promiscuous target selection is shared [49^••^]. As flexible AT-rich integration sites are also common in other conjugative transposons [52], their transposases may also rely on DNA distortions. Interestingly, the integron integrase, another mobile DNA-related TR that catalyses reshuffling of bacterial virulence and resistance gene cassettes under stress conditions, uses another peculiar mechanism to recognize diverse DNA sites. It interacts with flipped-out DNA bases at the cleavage site to promote recombination of various sequences with this feature [53]. Thus, distinct DNA deformations can be used in transposases and mobile DNA TRs to establish relaxed target specificity, which is the key for their spreading to a large range of bacteria.

## Conclusion

Despite the profound impact of transposons on genomes, their molecular mechanisms have remained poorly understood. Recent structures of transposase-DNA complexes and related cellular machineries have now exposed remarkable insights, highlighting a common role of DNA acrobatics in executing all reaction steps. Transposases from unrelated families bend, unwind and melt their DNA substrates to set off cleavage, deform DNA strands to complete excision or sharply bend target DNA for integration. These DNA acrobatics serve several purposes in transposition. Firstly, distortions help to bring different DNA strands into the transposase active site for sequential cleavage and re-joining. Secondly, DNA bending allows to stock up energy and drive isoenergetic strand exchange reactions forward. Thirdly, release of strained DNA conformations promotes product escape from the transposase active site, thus rendering the reactions irreversible.

Surprising architectural similarities of different transpososomes and their shared preference for flexible DNA sequences support a general involvement of DNA deformations in transposition. Consistently, accessory DNA bending proteins (e.g. IHF, Xis and HMGB) have been shown to promote the reactions of several DDE and Y-transposases [19^•^,32^•^,^54^,^55^]. Yet, we currently only understand a small fraction of the stunning diversity of transposition. From dozens of transposon families [14] only few have been structurally characterized, and the exact extent to which DNA deformations and their functional impact are conserved remains to be unraveled. Future studies of distinct transposon families at various reaction steps will be instrumental to address these questions.

Notably, transposons are related to key cellular machineries [5,6], integrases of CRISPR arrays (i.e. Cas1) [56,57], bacteriophages [40,41], retrotransposons, and endogenous and pathogenic retroviruses [58]. For several of these, recent studies highlighted mechanistic parallels, again showcasing an integral role of DNA acrobatics in their functions [19^•^,20^••^,^30^,^31^,32^•^,37^••^]. It will be interesting to explore this phenomenon in various biological settings to better understand its rules and global scope in genome rearrangements. As transposases provide valuable genetic tools, understanding their workings will also help to develop advanced technologies for research and biomedicine.

## Conflict of interest statement

Nothing declared.

## References and recommended reading

Papers of particular interest, published within the period of review, have been highlighted as:

• of special interest
•• of outstanding interest
